# Acute Effects of Split Pea-Enriched White Pan Bread on Postprandial Glycemic and Satiety Responses in Healthy Volunteers—A Randomized Crossover Trial

**DOI:** 10.3390/foods11071002

**Published:** 2022-03-29

**Authors:** Ronak Fahmi, Heather Blewett, Jo-Ann Stebbing, Nancy Olson, Donna Ryland, Michel Aliani

**Affiliations:** 1 Department of Food and Human Nutritional Sciences, University of Manitoba, Winnipeg, MB R3T 2N2, Canada; fahmir@myumanitoba.ca (R.F.); hblewett@sbrc.ca (H.B.); donna.ryland@umanitoba.ca (D.R.); 2 The Canadian Centre for Agri-Food Research in Health and Medicine (CCARM), 351 Taché Ave, Winnipeg, MB R2H 0G1, Canada; 3 Morden Research and Development Centre, Agriculture and Agri-Food Canada, Morden, MB R6M 1Y5, Canada; jstebbing@sbrc.ca (J.-A.S.); nolson@sbrc.ca (N.O.)

**Keywords:** split yellow pea, white bread, human trial, glycemic response, insulin, satiety, acceptability

## Abstract

Pulse consumption has been associated with reduced postprandial glucose response (PPGR) and improved satiety. The objective of this study was (i) to investigate the effects of fortifying white pan bread with split yellow pea (*Pisum sativum* L.) flour on PPGR and appetite-related sensations, and (ii) to determine whether Revtech heat processing of pea flour alters the postprandial effects. A randomized controlled crossover trial was performed with 24 healthy adults. Participants consumed 50 g available carbohydrate from bread containing 20% pea flour that was untreated (USYP), Revtech processed at 140 °C with no steam (RT0%), Revtech processed at 140 °C with 10% steam (RT10%), or a control bread with 100% white wheat flour (100%W). Blood samples were analyzed for glucose and plasma insulin at 0, 15, 30, 45, 60, 90, and 120 min post-meal. Appetite sensations and product acceptability were measured using visual analogue and 9-point hedonic scales. Results showed no significant difference in the postprandial glucose and insulin responses of different bread treatments. However, pea-containing variants resulted in 18% higher fullness and 16–18% lower hunger, desire to eat, and prospective food consumption ratings compared to 100% W. No differences in the aroma, flavor, color, and overall acceptability of different bread products were observed. This trial supports using pea flour as a value-added ingredient to improve the short-term appetite-related sensations of white pan bread without affecting the overall acceptability.

## 1. Introduction

The burden of obesity is rapidly increasing in Canada and throughout the world [1,2]. About 13% of the world’s adult population (more than 650 million) are clinically obese [2]. This number reaches 26.7% of the population in Canadian adults [3]. Obesity is the leading risk factor for serious chronic health conditions such as Type 2 diabetes mellitus (T2DM) [4]. The estimated prevalence of T2DM in Canada is currently more than 3.4 million individuals (10% of the population) and is projected to reach 4.4 million by 2030 [5]. The direct healthcare cost of diabetes alone was estimated at around CAD 3.8 billion in 2020 [5].

Dietary interventions appear to play a critical role in managing and preventing diabetes by optimizing postprandial glycemic control and maintaining healthy body weight [6,7]. Classifying carbohydrate-containing foods based on their immediate impact on postprandial glycemia was first established by Jenkins et al. (1981) [8]. Accordingly, high glycemic index (GI ≥ 70) foods are digested, absorbed, and metabolized faster, resulting in substantial fluctuations in blood glucose levels compared to foods with moderate (GI = 56–69) or low glycemic index (GI ≤ 55) [8]. A plethora of studies have shown the associations between low dietary GI and reduced risk of chronic diseases in general [9,10,11] and obesity [12,13], and T2DM in particular [14,15,16,17,18,19,20]. Replacing high GI diets with low GI foods is recommended by the Canadian Diabetes Association [21].

Pulses, the edible seeds from leguminous plants, including lentils, chickpeas, dry beans, and dry peas [22], are promising foods that could be used in dietary strategies to manage obesity and diabetes. Pulses are high in protein, dietary fiber, and slow-release carbohydrates [23,24], which contribute to the low GI of the food through increased viscosity, slowed digestibility, delayed gastric emptying, and slowed intestinal glucose absorption [25,26]. Several studies have linked the consumption of pulse-based foods to lower postprandial glycemic response and body weight. A meta-analysis of 41 randomized controlled trials published from 1981 to 2007 revealed that pulse consumption improved markers of longer-term glycemic control, including postprandial glucose levels, insulin responses, and hemoglobin A1c (HbA1c) in both healthy and diabetic individuals [27]. Likewise, a systematic review by Marinangeli (2016) [28] analyzed 29 acute clinical studies from 1980 to 2012 and concluded that substituting highly digestible carbohydrate foods with 1 cup of whole cooked pulses results in a significant decrease in the acute postprandial glycemic response at a magnitude that meets or exceeds Health Canada’s 20% threshold [29]. The favorable nutritional profile of pulses also plays an important role in weight management and satiety by reducing appetite, food, and energy intake [24,30]. Accordingly, Li et al. (2014) investigated the acute effects of pulse intake on the postprandial satiety response in a meta-analysis combining nine studies and 126 participants. Results indicated a 31% increase in satiety following dietary pulse ingestion compared to control meals in healthy participants [31].

Yellow pea (*Pisum sativum* L.) is one of the most important pulse crops widely cultivated in Canada, with 4.9 million metric tons total production in 2020 [32]. Although peas are relatively inexpensive and plentiful within the marketplace, their frequency of consumption remains the lowest among Canadians [33]. Like other pulses, yellow peas have a low glycemic index [8,34] and are rich sources of high-quality protein, resistant starch, fiber (both soluble and insoluble), vitamins, minerals, and polyphenols [35,36,37,38,39]. Compared with cereal grains such as wheat, yellow peas have twice the amount of protein and significantly higher fiber content. Additionally, peas and pea flour are high in lysine and can complement wheat proteins by providing balanced sources of amino acids [39]. The low cost and high-quality protein of yellow pea make it a worthy candidate for developing value-added functional products in the food industry. Incorporating pea ingredients into wheat-based bakery products is one of the cost-effective, viable strategies to improve the overall nutritional quality of such products [40].

The effects of pea and pea fractions in improving the postprandial glycemic responses are well documented [40,41,42]. A study by Dodd et al. (2011) showed a reduced overall glycemic index in staple meals such as spaghetti, potato, and rice due to adding peas to the meals [43]. Likewise, Marinangeli et al. (2009) reported a significant decrease in the blood glucose levels of 19 healthy participants who consumed biscotti developed with 100% whole pea flour compared to the control with 100% wheat flour. However, the same study indicated no significant difference in the glucose iAUC values when whole yellow pea flour was reformulated into banana bread or pasta. This was attributed to the differences in the food matrix and the effect of processing and cooking on the glycemic lowering effects of whole yellow pea flour as a pulse ingredient [44]. In another single-blind crossover trial conducted by Marinangeli and Jones (2011), both whole pea flour and fractionated pea flour (hulls only) reduced fasting insulin and insulin resistance in overweight persons (n = 23) with hypercholesterolemia [45]. A later study by Smith et al. (2012) observed a significant decrease in the blood glucose concentrations of healthy participants (n = 19) who consumed pea protein (10–20 g) as a part of their meal (tomato soup) compared to the control [46]. Adding a combination of pea protein and hull fiber to the meal (noodles and tomato) was also influential in attenuating the blood glucose levels in healthy consumers (n = 15) [47]. Considering the favorable GI properties, fairly abundant supply and low cost of pea products, they can be further explored as possible functional ingredients in reformulating and developing value-added products with improved health benefits, such as fortified bread. The present study focused on the effect of the incorporation of pea flour into wheat-based bread.

Bread has long been an essential part of the typical diet in Canada and around the world. The retail sale of bread is anticipated to reach CAD 4.2 billion in 2022, accounting for nearly two-thirds (64%) of the bakery market in Canada [48]. White bread is one of the most popular types of wheat-based bread [49]; however, it is classified as a high-glycemic food with an average GI of 71 [50]. One of the commonly proposed dietary strategies to improve the postprandial glycemic response and the overall nutritional quality is to incorporate pulse flours into wheat bread production. A study by Hall et al. (2005) showed that replacing 10% of the wheat flour with pulse flour (Australian lupin) led to a significant reduction in the GI of the white bread and a trend (*p* = 0.068) towards a lower glucose iAUC for blood glucose concentration in 11 healthy participants at 30 min post-consumption [51]. Likewise, a short-term study by Johnson et al. (2005) reported a trend (*p* = 0.087) towards reduced glycemic response in healthy subjects (n = 11) who consumed bread fortified with 23.4% chickpea flour as part of their breakfast meal compared to the control with 100% wheat bread [52]. A later work conducted by Zafar et al. (2015) recorded a significant decrease in the glucose iAUC in 13 healthy participants due to the supplementation of whole wheat bread with 35% chickpea flour [53].

Despite the high nutritional value and associated health benefits of pulses, including peas [27,28,31], their addition to staple foods such as bread remains challenging due to the unfavorable sensory characteristics and low consumer acceptability of the resulting products. Heat treatments such as roasting, blanching, micronization, and steam heating have shown to be effective in enhancing both the nutritional and sensory profiles of pulse-derived ingredients and legumes in general [54,55,56,57,58,59,60]. Our earlier work reported the efficacy of Revtech thermal processing in reducing the beany off-flavor in split yellow pea flour [61] and improving the consumer acceptability of white pan bread fortified with 20% pea flour [62].

Revtech is an emerging heating technology gaining in popularity as a successful means for the thermal processing of dry food ingredients. The system includes a combination of electrical heating and continuous vibrational transport of raw materials confined in a stainless-steel spiral that results in rapid, uniform, and highly efficient heat transfer [63]. The technology was approved by the Food and Drug Administration (FDA) and can be used for pasteurization, sterilization, drying, roasting, and other heat treatments of various products, including grains, seeds, legumes, spices, nuts, starch, and flour. In addition to the efficient heat transfer to the product and even treatment, Revtech is an environmentally friendly technology with low energy consumption and low-to-zero gas emission [63].

Incorporating pea flour in staple foods such as wheat-based bread is an effective means to promote pulse consumption while reinforcing the health benefits and nutritional density of such products. However, a limited number of clinical studies have investigated split yellow pea flour and the effects of pulse-enriched bread on the postprandial glycemic or satiety responses in healthy consumers. Therefore, the aim of the current study was (1) to investigate the effect of fortifying white bread with 20% pea flour on the postprandial glycemic and insulinemic responses, as well as the appetite-related sensations and acceptability of developed products; (2) to determine whether Revtech processing, with and without the addition of steam, alters the postprandial effects of fortified bread. The present study considered Health Canada’s guidance document for the postprandial glycemic response [29] and satiety health claims on food [64].

## 2. Materials and Methods

### 2.1. Subjects

**Figure 1:**
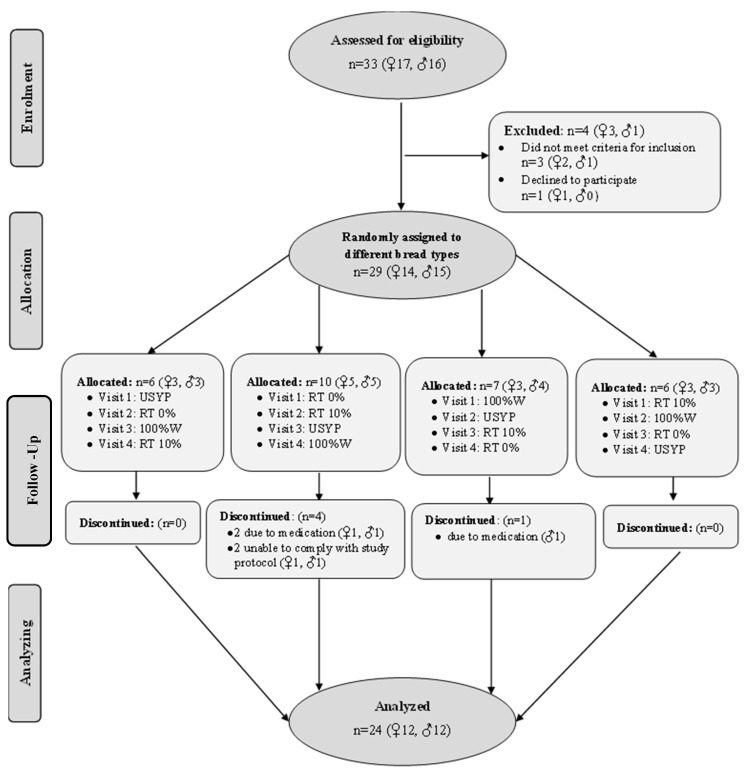
Flowchart illustrating the selection procedure of study subjects. Notes: n, number of participants; ♀, female; ♂, male; USYP, bread containing 80% wheat flour and 20% untreated pea flour (no Revtech process); RT0%, bread containing 80% wheat flour and 20% pea flour Revtech processed at 140 °C with no steam; RT10%, bread containing 80% wheat flour and 20% pea flour Revtech processed at 140 °C with 10% steam; 100%W, bread containing 100% wheat flour (control).

Healthy adults aged 18–40 years with a body mass index (BMI) of 18.5–30.0 kg/m^2^ were eligible and recruited through advertisements posted around the University of Manitoba, St. Boniface Albrechtsen Research Centre, in classified e-newsletters, student group emails, and on site-affiliated websites. Exclusion criteria included pregnancy or breastfeeding, medical history of diabetes (fasting blood glucose ≥ 6.1 mmol/L, HbA1c ≥ 6.0%, or use of insulin or oral medication to control blood sugar); medical history of cardiovascular disease; systolic blood pressure > 140 mmHg or diastolic blood pressure > 90 mmHg; fasting plasma total cholesterol > 7.8 mmol/L; fasting plasma HDL < 0.9 mmol/L; fasting plasma LDL > 5.0 mmol/L; fasting plasma triglycerides > 2.3 mmol/L; major surgery within the past 3 months; medical history of inflammatory disease (i.e., systemic lupus erythematosus, rheumatoid arthritis, psoriasis) or use of any corticosteroid medications within 3 months; medical history of liver disease or liver dysfunction (defined as plasma AST or ALT ≥3 times the upper limit of normal); medical history of kidney disease or kidney dysfunction (defined as blood urea nitrogen and creatinine ≥3 times the upper limit of normal); presence of a gastrointestinal disorder, daily use of any stomach acid-lowering medications or laxatives (including fiber supplements) within the past month or antibiotic use within the past 6 weeks; active treatment for any type of cancer within 1 year prior to study start; shift worker; smoking, use of tobacco or a nicotine replacement product (within the past 3 months); allergies to peas or wheat; aversion or unwillingness to eat study foods; consuming > 4 servings of pulses per week; use of any prescription or non-prescription drug, herbal or nutritional supplement known to affect glycemia or appetite; participation in another clinical trial, current or in the past 4 weeks; unstable body weight (defined as >5% change in 3 months) or actively participating in a weight loss program. All subjects had signed the informed consent forms prior to initiating the study. Participant flow is depicted in Figure 1.

### 2.2. Ethics and Study Design

The present study took place at the I. H. Asper Clinical Research Institute at St. Boniface Hospital in Winnipeg, Manitoba, Canada, from April 2019 to December 2019. The study was carried out according to the Helsinki declaration and was approved by the University of Manitoba Biomedical Research Ethics Board (HS21603 (B2018:026)), the St. Boniface Hospital Research Review Committee (RRC/2018/1748) and the Agriculture and Agri-Food Human Research Ethics Committee (2018-D-006 Blewett). This study was registered on the clinical trial registry website (http://www.clinicaltrials.gov; accessed on 18 January 2022) with the identification number of (ID # NCT03506932).

This trial followed a single-site, randomized, controlled, crossover design, in which participants received in random order 1 of 4 treatments per visit. The order of the treatments was generated by computer using a random sequence generator program available at www.random.org (accessed on 18 January 2022). The participants were required to attend four study visits, each lasting for approximately 2.5 h, and were separated by a 3-to-14-day washout period. The experimental procedures and treatments are described in Section 2.5.

### 2.3. Bread Products

Straight grade wheat flour, milled from a grist of Canadian Western Spring Wheat (CWRS) and English wheat, was provided by Warburtons (Bolton, UK) from Nelstrop William & Co. Ltd. (Stockport, UK). Split yellow pea (*Pisum sativum* L.) flour was obtained from Avena Foods Limited (Portage la Prairie, MB, Canada). Pea flour was processed at 140 °C with steam (10%) and without in pilot-scale RT equipment (Revtech Process Systems, Loriol-sur-Drôme, France) located at Campden BRI (Chipping Campden, UK). The RT system was operated with a motor angle of 40 degrees, residence time of 4 min, vibrational frequency of 740 rpm, and flow rate of 150 kg/h. The treatment parameters (heat/steam) were selected based on preliminary trials carried out by the Canadian International Grains Institute (Cigi) as described previously by Fahmi et al. (2019) [62]. Flours and blends prepared using 20% pulse flour and 80% wheat flour were stored in covered food-grade plastic pails (20 L) at 22 °C, 50% relative humidity (RH) until baked. 

All experimental pan breads were developed and prepared by Cigi using its standard formulation and processing steps as described in our earlier study [62]. The following four pan breads were used in this study: (1) USYP, 80% wheat flour and 20% untreated pea flour (no Revtech process); (2) RT0%, 80% wheat flour and 20% pea flour Revtech processed at 140 °C with no steam; (3) RT10%, 80% wheat flour and 20% pea flour Revtech processed at 140 °C with 10% steam; and (4) 100%W, 100% wheat flour (control).

All bread products were shipped to the I. H. Asper Clinical Research Institute at St Boniface Hospital (Winnipeg, MB, Canada), where they were stored at −20 °C until use in the clinical trial, approximately 3–12 months.

### 2.4. Nutrient Analysis

Bread samples were analyzed for nutritional composition by proximate analysis conducted by Central Testing Laboratory Inc. (Winnipeg, MB, Canada) using standard methods. Moisture % (AOAC 930.15; AOAC, 2005a) [65], crude protein (%) (modification of AOAC 990.03; AOAC, 2005b) [66], fat% (AOCS AM 5-04; AOCS, 2017a) [67], crude fiber % (AOCS Ba6a- 05; AOCS, 2017b) [68], ash% (AOAC 923.03; AOAC, 2005c) [69], and total carbohydrate were calculated by difference (100—sum of protein, fat, ash, and moisture). Calories were obtained (cal/100 g) by calculation. Total sugar content (including monosaccharides and disaccharides) was determined by Intertek (Saskatoon, SK, Canada) according to the modified AOAC 980.13 [70] method. The total starch and resistant starch contents were measured by Intertek using AACC 76-13 (AACC, 2000) [71] and AACC 32-40.01 (AACC, 2002) [72] methods, respectively. The nutritional composition of different bread treatments (n = 1) is summarized in Table 1.

### 2.5. Study Protocol

**Figure 2:**
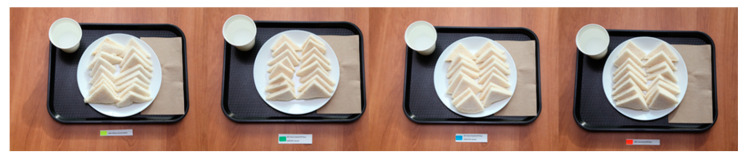
Fasting breakfast of different bread treatments, including one serving of test bread and 250 mL water.

Participants were scheduled to arrive in the morning following a 10–12 h overnight fast. Each breakfast meal consisted of one serving of test bread in random order (USYP, RT0%, RT10%, or 100%W) and 250 mL of distilled water (Figure 2). The slices of bread were removed from the freezer, crusts were cut off, and bread was placed in a Ziploc bag at room temperature to thaw for 20 min prior to consumption. The amount of each test bread was standardized to provide 50 g of available carbohydrate per serving. The nutritional composition of different bread treatments is outlined in Table 1. Participants were required to eat all the given food within 10 min. The day before their first visit, all participants were asked to complete a pre-visit food record and repeat the same food pattern before all subsequent visits. All subjects were instructed to avoid strenuous activity, alcohol consumption, and any foods containing pulses (beans, peas, chickpeas, lentils) the day before each clinic visit. If any significant deviations from their usual patterns were reported, the sessions were rescheduled. Capillary and venous blood collection was carried out over two hours, as outlined in Section 2.6. Participants were also asked to complete an appetitive visual analogue scale (VAS) and product acceptability questionnaire during their 2 hr clinic visit as described in Section 2.7 and Section 2.8, respectively.

### 2.6. Glucose and Insulin Measurements

For glucose analysis, fingertip capillary blood was collected at fasting and 15, 30, 45, 60, 90, and 120 min after the first bite of the test product according to the Clinical Laboratory Standard Institute (CLSI) guidelines. Blood glucose concentrations at each time point were measured at the Clinical Research Institute using a portable STAT STRIP glucometer (StatStrip Glucose, Nova Biomedical Waltham, MA, USA). All samples were analyzed in duplicate, and if they differed by more than 0.3 mmol/L, additional samples were analyzed until two readings were within 0.3 mmol/L of each other [74].

For insulin analysis, venous blood was collected into lithium heparin tubes through an indwelling catheter at 0, 15, 30, 45, 60, 90, and 120 min. Blood samples were inverted 8–10 times and centrifuged immediately at 1300 g for 10 min at 21 °C (Thermo Scientific, Sorvall ST 16R centrifuge) to separate plasma. The resulting plasma was then aliquoted into 600 µL Eppendorf tubes and stored at –80 °C until analyzed. Insulin concentrations at each time point were measured using the immunoassay analyzer COBAS e411 (Roche Diagnostics, Indianapolis, IN, USA).

Incremental areas under the curve (iAUC) for blood glucose and plasma insulin for different test breads were calculated using methods described by Brouns et al. (2005) [73]. The glycemic index (GI) values were calculated by dividing the iAUC for each test bread by the iAUC of standard food (100%W) and multiplying the ratio by 100 and then by 0.7 using the following formula [75]:

Glycemic index = [(iAUC_test food_/iAUC_standard food_) × 100 × 0.7]

### 2.7. Satiety Assessment

The subjective perceptions of satiety were measured using visual analogue scales (VAS) at baseline (pre-meal) and 30, 60, 90, and 120 min after the first bite of the bread products. Accordingly, participants were asked to assess four appetite sensations, including hunger, fullness, desire to eat, and how much food they felt they could consume (prospective food consumption) at each time point. The VAS consisted of a 10 cm printed line anchored at each end with opposing extreme statements for each variable (for example, ‘not at all hungry’ to ‘extremely hungry’). Subjects were required to place a vertical mark anywhere along the scale that matched their perception of satiety at that particular time. The VAS ratings were converted to numerical scores by measuring the distance in centimeters from the far-left statement to the intersection of the marked line on the scale.

Incremental satiety responses for each bread variant were determined by considering the cumulative area under the curve of five time points (30 min intervals) over the whole 120 min using above baseline trapezoidal calculations [51,52].

### 2.8. Sensory Analysis

Participants were asked to complete an acceptability questionnaire at the 15 min time point to rate their opinion of the bread products with respect to color, aroma, flavor, texture, and overall acceptability. Each parameter was evaluated using a 9-point hedonic scale where 1 = dislike extremely; 2 = dislike very much; 3 = dislike moderately; 4 = dislike slightly; 5 = neither like nor dislike; 6 = like slightly; 7 = like moderately; 8 = like very much; and 9 = like extremely. Data were also collected using another measure of acceptability to determine how often the participants would eat each bread product if available outside the study (frequency of eating). One of the following nine descriptors was selected: 1 = I would eat this only if forced; 2 = I would eat this if there were no other food choices; 3 = I would hardly ever eat this; 4 = I don’t like this but would eat it on occasion; 5 = I would eat this if available but would not go out of my way; 6 = I like this and would eat it now and then; 7 = I would frequently eat this; 8 = I would eat this very often; 9 = I would eat this every opportunity I had. Subjects were required to checkmark ✓ under the response that best corresponded to their feelings on each product. Participant ratings for each product were compiled at the end for statistical analysis.

### 2.9. Statistical Analysis

The sample size was determined based on power analysis for a within-subject design from the results of a similar study that investigated the effect of fiber on the postprandial glycemic response and resulted in a standard deviation of 19 [76]. A sample size of 24 was calculated to detect a 20% difference in the glucose iAUC at a power of 0.8 and a significance level of *p* = 0.05.

Data for glucose, insulin, and satiety postprandial responses were analyzed using PROC MIXED repeated measures with bread treatment as a fixed effect and subject as a random variable using the SAS system version 9.4 (SAS Institute Inc., Cary, NC, USA). Three-way repeated-measures ANOVA using the PROC MIXED model was applied to test for the effects of time, treatment, sex, and their interactions (time by treatment, time by sex, and treatment by sex) for the glucose and plasma insulin concentrations, as well as the average appetite scores over 120 min. For the GI and iAUC, analysis of variance using PROC GLM with the following sources of variation was included in the model: treatment (100%W, USYP, RT0%, RT10%); sex (male, female); order; ID (individual); treatment by sex (T × S); and treatment by order (T × O). Differences among means were determined by Lsmeans (adjusted for multiple comparisons using Tukey). One-way ANOVA was used to compare the sensory analysis scores of different bread products using SPSS software version 26.0 (SPSS Inc., Chicago, IL, USA). Tukey tests were used for all post hoc analysis. Differences were considered statistically significant with *p* < 0.05. All results are presented as means with their standard error of the mean. 

Partial least squares regression (PLS-R) was applied to explore the associations between the postprandial glycemic and insulinemic responses (Y variable) and the other measured parameters (X variables including the appetite-related sensations and sensory attributes) using mean values for all variables (XLSTAT, version 19.4; Addinsoft, Paris, France).

## 3. Results

### 3.1. Subject Characteristics

Of the 29 participants who took part in the study, 24 healthy subjects with a mean BMI of 22.4 ± 1.4 kg/m^2^ and an average age of 24.5 ± 4.3 years completed the entire four-session trial. Three individuals discontinued the study for medical reasons, while two were unable to comply with the study protocol. After dropouts, a total number of 12 male and 12 female participants completed the experiment. Further details on the participants’ ethnicity and baseline characteristics are outlined in Table 2.

### 3.2. Blood Glucose and Plasma Insulin Responses

**Figure 3:**
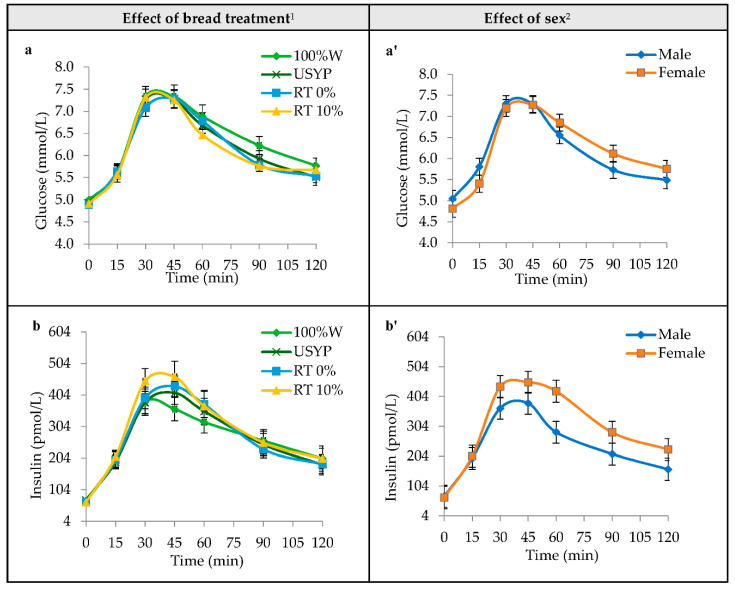
Postprandial blood glucose (**a**,**a’**) and plasma insulin (**b**,**b’**) responses in healthy adults as affected by bread treatments (**left column**) and sex (**right column**) at specific time points over 120 min. **Notes:** Treatments were USYP, bread containing 80% wheat flour and 20% untreated pea flour (no Revtech process); RT0%, bread containing 80% wheat flour and 20% pea flour Revtech processed at 140 °C with no steam; RT10%, bread containing 80% wheat flour and 20% pea flour Revtech processed at 140 °C with 10% steam; 100%W, bread containing 100% wheat flour (control). All bread treatments included 50 g available carbohydrate. ^1^ Values are means (n = 24 participants/treatment), with their standard errors represented by vertical bars. ^2^ Values are means (n = 12 participants/sex), with their standard errors represented by vertical bars.

The average glucose concentrations at specific time points (0, 15, 30, 45, 60, 90, and 120 min) for each bread treatment (100%W, USYP, RT0%, and RT10%) in healthy subjects are presented in Figure 3a. Blood glucose concentration was significantly affected by time (*p* ≤ 0.0001), but not treatment (*p* = 0.16) or time by treatment interaction (*p* = 0.37). Glucose levels peaked from the baseline of 4.9 mmol/L to reach 7.3 mmol/L at 30 to 45 min for different treatments and declined gradually towards the initial value by 120 min.

The average blood glucose response in male and female participants is shown in Figure 3a’. There was no significant effect of sex (*p* = 0.92), treatment by sex (*p* = 0.91), or time by sex (*p* = 0.08) interactions on glucose levels.

The incremental insulinemic response to different bread variants over a period of 2 h is illustrated in Figure 3b. Like the blood glucose pattern, insulin levels increased from baseline (average of 67.8 pmol/L) following consumption, peaking at around 30 min for 100%W (381 pmol/L) and at 45 min for USYP (410 pmol/L), RT0% (431 pmol/L), and RT10% (461 pmol/L) products. However, these differences were not statistically significant. The insulin concentration was significantly affected by time (*p* ≤ 0.0001), but not treatment (*p* = 0.22) or time by treatment interaction (*p* = 0.07).

Regardless of the treatment, females showed higher insulin concentrations compared to their male counterparts (Figure 3b’). The average insulin response concentration peaked at 45 min with 381 and 452 pmol/L for male and female participants, respectively. However, the mentioned differences did not reach statistical significance (*p* = 0.18) at any of the time points in the insulin response over the 120 min. The interaction of time by sex (*p* = 0.09) was not significant either.

The cumulative incremental area under the curve calculated over 120 min (iAUC) for blood glucose and plasma insulin responses and the glycemic index in different bread variants are presented in Table 3. Although pea-containing bread samples (USYP, RT0%, and RT10%) showed 6% to 11% lower blood glucose iAUC values compared to the control wheat bread (100%W), these differences did not reach statistical significance (*p* = 0.59). Similar results were obtained for the insulin iAUC value (*p* = 0.12) and the glycemic index (*p* = 0.39).

Aside from the treatment, our results demonstrated a significant effect of sex on the glucose and insulinemic responses in male and female participants (Table 3). Females showed 26% (*p* = 0.0002) higher blood glucose (iAUC) and 41% (*p* < 0.001) higher plasma insulin (iAUC) responses.

The effect of order (*p* < 0.0001) and ID (*p* < 0.0001) on the glucose and insulin iAUC values was significant, which could be attributed to the individual differences in producing different concentrations of glucose and insulin in response to consumed bread (Table 3). However, neither the treatment by sex (*p* ≤ 0.91) nor treatment by order (*p* = 0.89) interactions were significant.

Further information on the postprandial glucose and insulin responses as affected by time and treatments in both sexes is depicted in the Supplementary Materials (Figures S1–S4) using box plots.

### 3.3. Satiety Response

**Figure 4:**
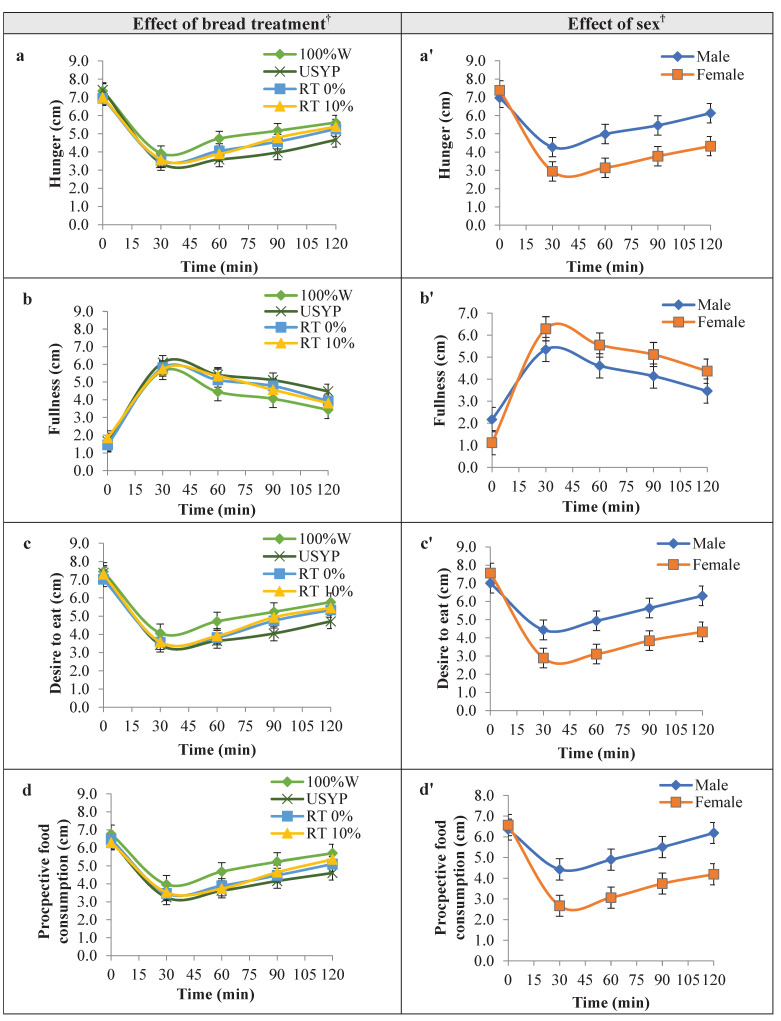
Average postprandial appetite sensation ratings ((**a**,**a’**), hunger; (**b**,**b’**), fullness; (**c**,**c’**), desire to eat; (**d**,**d’**), prospective food consumption) in healthy adults as affected by bread treatments (**left column**) and sex (**right column**) at specific time points over 120 min. **Notes:** Treatments were USYP, bread containing 80% wheat flour and 20% untreated pea flour (no Revtech process); RT0%, bread containing 80% wheat flour and 20% pea flour Revtech processed at 140 °C with no steam; RT10%, bread containing 80% wheat flour and 20% pea flour Revtech processed at 140 °C with 10% steam; 100%W, bread containing 100% wheat flour (control). ^1^ Values are means (n = 24 participants/treatment), with their standard errors represented by vertical bars. ^2^ Values are means (n = 12 participants/sex), with their standard errors represented by vertical bars.

Comparisons of postprandial satiety responses for different bread products (100%W, USYP, RT0%, and RT10%) are summarized in Figure 4a–d. The average appetite scores for hunger, desire to eat, and prospective food consumption decreased from the baseline after the consumption of all four bread variants up to 30 min, then slowly rose over time. On the other hand, the average fullness ratings climbed sharply at 30 min and declined steadily thereafter without reaching the baseline in all bread. There were no statistically significant differences between the bread treatments at any time points in the incremental satiety responses over 2 h.

Figure 4a’–d’ illustrates the effect of sex on the average appetite sensation ratings. Though female participants showed higher fullness and lower hunger, desire to eat, and prospective consumption ratings compared to the male participants, neither of the differences in the appetite sensations between the two sexes reached statistical significance at any of the time points.

The three-way ANOVA repeated measures followed by Tukey post hoc test showed time (*p* < 0.0001) effects for all appetite sensations. The treatment effect was significant for fullness (*p* = 0.04), desire to eat (*p* = 0.02) and prospective food consumption (*p* = 0.01), but not hunger *p* = 0.09). Time by treatment interactions were found not significant for any of the sensations (hunger, *p* = 0.19; fullness, *p* = 0.17; desire to eat, *p* = 0.27; prospective food consumption, *p* = 0.36). The effect of sex, on the other hand, was significant for the majority of mentioned perceptions (hunger, *p* = 0.04; desire to eat, *p* = 0.03; prospective food consumption, *p* = 0.03) except fullness (*p* = 0.38). A time by sex interaction was also present (fullness, *p* = 0.04; desire to eat, *p* = 0.01; prospective food consumption, *p* = 0.003), except hunger (*p* = 0.05) (Figure 4).

The cumulative incremental area under the curve (iAUC) for different appetite sensation variables is calculated and summarized in Table 4. Bread fortified with pea flour (USYP, RT0%, and RT10%) had lower ratings for hunger, desire to eat, and prospective food consumption, and higher fullness sensation compared to control white bread (100%W). The lowest iAUC values belonged to USYP bread with 16%, 17% and 18% decrease in hunger, desire to eat, and prospective consumption ratings, respectively. Conversely, USYP resulted in an 18% increased fullness rating compared to 100%W. Interestingly, the prospective consumption was significantly reduced in all pea-enriched bread.

Regardless of the treatment, there were significant differences between the satiety perceptions iAUC values in male and female participants (Table 4). Males showed 36%, 38%, and 44% higher hunger, desire to eat, and prospective consumption ratings, respectively, whereas the mean iAUC for fullness was significantly higher (17%) in female participants.

The effect of both order (*p* < 0.0001) and ID (*p* < 0.0001) was significant for hunger, fullness, desire to eat, and prospective food consumption values, possibly due to the differences in satiety perceptions in different individuals for each bread treatment (Table 4). However, there was no effect for treatment by sex or treatment by order interactions on any of the appetite sensation variables.

### 3.4. Acceptability of Bread Products

Results comparing sensory characteristics of different bread treatments are summarized in Table 5. Accordingly, pea-enriched samples were not significantly different in acceptability for aroma, flavor, color, and overall acceptability compared to control wheat bread (100%W). There were no significant differences between the three pea-containing variants either. The only sensory differences noted were for texture, where acceptability for RT0% was 22% lower (*p* = 0.002) than 100%W (7.3 ± 0.3). All bread samples were rated at or above 5.7 (representing ‘like slightly’ on the 9-point scale) for all sensory parameters.

As for the frequency of eating, no significant differences between the three pea-containing products and 100%W were observed. All bread variants rated at or above 5.9 (‘I like this and would eat it now and then’), except for RT0%, where the mean value was slightly lower (5.4 ± 0.4).

### 3.5. PLS-R

**Figure 5:**
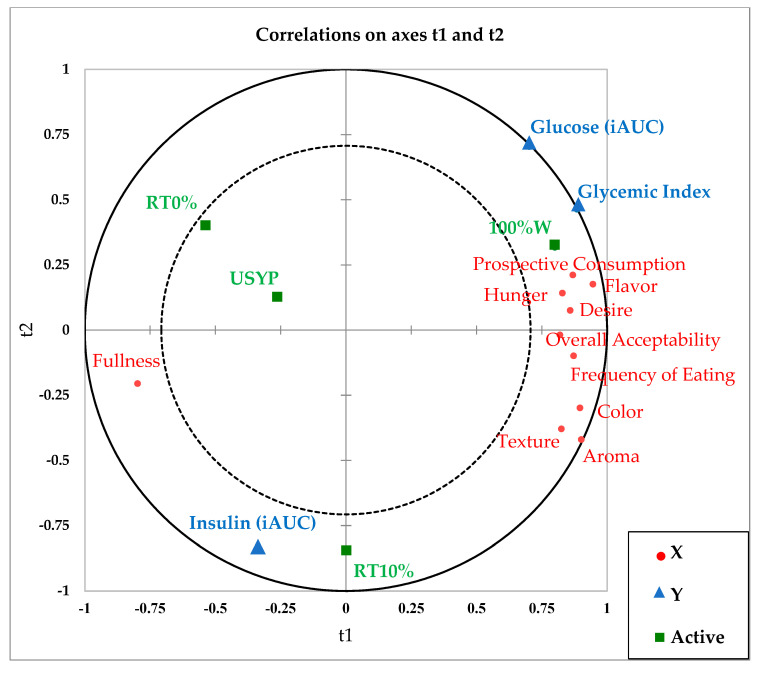
Partial least squares regression plot (PLS-R) for four bread treatments (observations) showing the correlation between X (t1) and Y (t2) variables where: **Notes:** X variables ● = Acceptability attributes (aroma, flavor, color, texture, frequency of eating, overall acceptability) and satiety sensations (hunger, fullness, desire to eat, and prospective food consumption rating scales). Y variable ▲ = Postprandial blood glucose (iAUC, mmol/L × min), and plasma insulin (iAUC, pmol/L × min) responses, and glycemic index. Observations ■ = USYP, bread containing 80% wheat flour and 20% untreated pea flour (no Revtech process); RT0%, bread containing 80% wheat flour and 20% pea flour Revtech processed at 140 °C with no steam; RT10%, bread containing 80% wheat flour and 20% pea flour Revtech processed at 140 °C with 10% steam; 100%W, bread containing 100% wheat flour (control).

Potential correlations between the acceptability attributes (aroma, flavor, color, texture, overall acceptability, and frequency of eating), the appetite-related sensations (hunger, fullness, desire to eat, and prospective food consumption rating scales), and the postprandial glycemic and insulinemic responses of different bread products are exhibited in the partial least squares regression (PLS-R) plot in Figure 5. The four bread variants were grouped separately in different areas on the plot, denoting their different characteristics. The control wheat bread (100%W) was located fairly close to the glucose iAUC and glycemic index at the top right quadrant. The majority of satiety sensation variables, including hunger, desire to eat, and prospective consumption, were clustered on the upper right quadrant near 100%W. The sensory attributes were also grouped on the far right of the plot in close proximity with 100%W, indicating its correlation with acceptability. RT0% and USYP, on the other hand, were in the opposite left quadrant far from the acceptability parameters, and therefore would be considered less acceptable. Close to these two pea-containing bread samples was the fullness appetite sensation. RT10% appeared in the bottom quadrant far from other bread variants and relatively close to the insulin iAUC value.

## 4. Discussion

Nowadays, peas and pea products, including flour, are receiving significant interest in the food industry as value-added ingredients that can be used in formulating low glycemic, functional foods with improved nutritional properties. This study examined the effect of replacing wheat flour in white pan bread with 20% pea flour (USYP, RT0%, RT10%) on the postprandial glycemic, insulinemic, and satiety responses in healthy subjects.

Though canned and home-cooked yellow peas were reported to be effective in lowering the post-meal glycemia in earlier studies [40,41,77,78], our results showed that fortifying the white pan bread with 20% pea flour did not affect the glycemic response. The consumption of none of the pea-enriched bread variants (USYP, RT0%, RT10%) led to a statistically significant reduction in the postprandial glucose levels (iAUC) compared to the control (100%W). There are several possible explanations for the obtained results. First, the amount of yellow pea flour (20%) used in our study was not sufficient to reduce the GI of the product and the corresponding glycemic response. This was in accordance with Johnson et al. (2005), who reported no significant decrease in the short-term glycemic response as a result of adding 23.4% chickpea flour (regular and extruded) to white pan bread [52]. Likewise, Zafar et al. (2015) observed no alteration in the blood glucose levels of healthy participants who consumed whole wheat bread enriched with 25% chickpea flour [53]. However, the same study by Zafar et al. (2015) showed that increasing the amount of chickpea flour from 25% to 35% significantly decreased the glucose iAUC, indicating the importance of incorporation level in determining the physiological effect of pulse-derived ingredients [53]. Second, it is possible that split yellow pea flour does not produce the same reduction rate in post-meal blood glucose levels as do whole cooked pulses due to the differences in nutritional composition and matrix of the used pulse ingredients. Split yellow pea is prepared from whole peas that have undergone a milling operation to remove the hull (testa) and separate the cotyledons [79]. Pea hull fiber is a rich source of dietary fiber (75–80%) [80]. The effect of pea hull in lowering glycemic and insulinemic responses has been reported in earlier studies [45,47,81]. The fiber content in pea-enriched bread samples was 0.7–1.3% (Table 1). However, the analysis of fiber in the current study was done on a crude basis, which may not have adequately captured all of the dietary fiber present in bread samples. The third reason could be related to the smaller particle size in pea flour. Dehulled split peas are processed into flour by further milling and particle size reduction [79]. This could be explained considering the larger surface area of flour particles, which increased the exposure to enzyme attack, leading to an increased starch digestion rate [82]. Fourth, it is possible that the glycemic load of 50 g of available carbohydrate (provided in each bread serving) overpowered the expected favorable metabolic effects of pulse flour enrichment. Finally, test breads in the current study were served alone, which does not represent a typical dietary pattern. Perhaps consuming the bread samples as a part of a mixed meal could have different synergistic or suppressive effects on the blood glucose response. Despite the mentioned explanations, further research into the effects of pea flour on postprandial glycemia in baked products is required.

Similar to the glycemic pattern, insulin levels increased from baseline following consumption of test bread, peaking at 30 to 45 min and returning toward the baseline before 120 min. No significant difference between 100%W and pea-containing bread (USYP, RT0%, RT10%) was observed at any time points. Insulin plays a vital role in regulating blood sugar levels. In addition to the carbohydrates load, the presence of rapidly digested proteins can also stimulate insulin secretion [83]. Accordingly, the insulin response is closely affected by the concentration of plasma amino acids such as arginine, leucine, isoleucine, valine, and phenylalanine [84,85].

Despite the higher protein levels in USYP (12.1 g), RT0% (12.4 g), and RT10% (12.6 g) compared to the 100%W (9.3 g), none of the pea-enriched bread led to a significantly increased insulin response (iAUC) compared to the control white wheat bread. Higher pea flour incorporation rates (>20%) that could translate into higher protein content may be required to reach a statistically different insulinemic response in fortified bread. An earlier study by Johnson et al. (2005) reported a significant hyperinsulinemic effect for breakfast that included white bread enriched with 24.3% chickpea flour compared to control white bread. However, the same study showed no difference in the insulinemic response when the extruded chickpea flour was applied [52]. This was attributed to the effects of the high temperature, high-pressure extrusion process on the chickpea protein [52]. Another study by Hall et al. (2005) demonstrated the hyperinsulinemic effect of adding 10% Australian lupin flour to white wheat bread eaten as part of a breakfast meal by healthy adults [51]. This was also attributed to the elevated protein content in the lupin-enriched bread compared to the white wheat bread [51]. The discrepancy in the insulinemic response between these studies may be due to the differences in the composition of pulse/legume flours, levels of substitution, the carbohydrate load, or the type of treatments where the test bread was served alone (like the present study) or as a part of a standardized mixed meal. Further studies investigating the putative effects of pea flour and pea-containing products on the insulinemic response appear warranted.

Interestingly, the addition of pea flour to standard white bread resulted in a considerable increase in the subjective perceptions of satiety. Participants who consumed pea-enriched breads (USYP, RT0%, and RT10%) reported a higher sense of fullness (13% on average) and less hunger (12% on average), desire to eat (12% on average), and prospective food consumption (14% on average) ratings than when they consumed the control (100%W). The favorable satiety response in the present study could be explained by the increase in protein content, which was 30–35% higher in pea-containing bread than the control. The peak incremental satiety scores in all bread variants were observed at 30 min after fasting. Pea protein has been shown to be effective in regulating the short-term food intake and satiety responses in earlier studies [46,77]. The maximum suppression of food intake by pea protein at 30 min was found to be in accordance with the peak in plasma amino acid contents following meal ingestion [46]. Calbet and MacLean (2002) argued that pea protein acts like whey protein in raising the concentration of plasma amino acids and the appetite-regulating hormones [84]. That said, our findings do contradict some earlier studies, which showed no statistically significant differences in the self-reported satiety for white pan bread fortified with 24.3%, 25%, and 35% chickpea flour compared to the control [52,53]. Enrichment of standard white pan bread with 10% lupin flour also showed no improvement in participants’ satiety [51]. The explanation for the lack of statistical significance in mentioned studies was attributed to their small sample size (n ≤ 13) that was insufficient (underpowered) to detect differences between different bread treatments.

Sensory evaluations revealed that all bread samples fortified with regular and Revtech heat-treated pea flours were comparable to the standard white wheat bread. These are very encouraging results as acceptance and preference of the sensory properties of reformulated food products are two of the major determinants of food choice [86]. There were no statistically significant differences in the aroma, flavor, and color acceptability between USYP, RT0%, RT10%, and the control 100%W. The overall acceptability for all bread variants was scored above 6 (defined as ‘like slightly’ on a 9-point hedonic scale), indicating the acceptability of the end products among the subjects. This was in accordance with our earlier study where 110 consumers rated overall acceptability for the same pea-enriched breads as ‘like slightly to like moderately’ (6.3 to 6.9) compared to control white bread ‘like moderately’ (7.2) [62]. Success in developing a pulse-fortified bread with no altered organoleptic properties and good sensory quality is an important finding as taste/flavor acceptability is a primary driver for consumer choices. Similar promising results were reported in a recent study by Paladugula et al. (2021), who investigated the sensory acceptability of pea-enriched bread at the level of 20% using 60 individuals [58]. Though the consumers were able to detect the differences between bread treatments, no statistically significant differences in the overall acceptability were observed between the control and pea-containing breads [58]. Comparable results were observed for chickpea flours (regular and extruded), where supplementation of white wheat bread at the level of 23.5% [52] and whole wheat bread at the level of 35% [53] showed no adverse effects on the palatability.

Overall, the PLS-R plot confirmed the positive association between glucose iAUC, glycemic index, and 100%W bread. On the other hand, an apparent dissociation was observed for USYP, RT0%, and RT10%. This was in line with the 8% (on average) decrease in blood glucose response in pea-containing bread samples compared to the control. Though neither of these differences reached statistical significance (ANOVA results), the effect of pulse addition on decreasing the postprandial glycemic response was noticeable from the PLS plot. Among different pea-enriched bread variants, the insulin iAUC was near RT10%. This could be explained by the 17% increase in plasma insulin concentrations in participants who consumed RT10% bread compared to the control. Perhaps the effect of heat treatment (in the presence of steam) on the amino acid profile or the bioavailability of other nutrients in RT10% played a role in these differences, which requires further investigation. Finally, and as expected, the sensory attributes were positively associated with 100%W, whereas pea-enriched samples were associated with increased fullness, as demonstrated in the PLS plot.

The results of the present study could be of interest to the food industry for developing pea-fortified bread products with improved nutritional quality and acceptable sensory properties.

## 5. Conclusions

In conclusion, this study provides evidence that split yellow pea flour can be used as a functional ingredient to improve white pan bread’s short-term appetite-related sensations without adversely affecting the overall acceptability and sensory characteristics of the reformulated bread. The favorable satiety response in pea-enriched bread products was attributed to their improved nutritional composition due to increased protein contents. However, the addition of pea flour to white bread, with or without Revtech heat treatments, failed to show any positive results in reducing the blood glucose and insulin concentrations at 20% fortification levels. This observation emphasizes the importance of assessing the postprandial properties of pulse-derived foods as a part of the food formulation and product development process, as the glycemic lowering effects of whole pulses do not necessarily translate to the processed pulse ingredients. Further work is required to investigate the effect of higher incorporation rates (>20%) of split yellow pea flour on the short-term postprandial glycemic and insulinemic responses. As efforts to promote pulse consumption continue, further research into the effect of pea flour and other pea-derived ingredients (such as protein isolate, fiber fractions, etc.), different heat treatments, varied substitution rates, and larger participant sample size are warranted to understand better the putative physiological effects of pea flour consumption in both healthy and diabetic populations.

## Figures and Tables

**Table 1:** Nutritional composition of bread treatments.

	Bread Treatments			
	100%W	USYP	RT0%	RT10%
Energy (Kcal)	255.7	268.6	265.8	275.2
Weight (g)	100.2	103.9	103.0	107.3
Energy density (kcal·g^−1^)	2.6	2.6	2.6	2.6
Available Carbohydrate (g) *	50.0	50.0	50.0	50.0
Protein (g)	9.3	12.1	12.4	12.6
Fiber (g)	1.0	0.7	0.8	1.3
Fat (g)	2.2	2.6	2.6	2.6
Ash (g)	3.3	2.9	3.1	2.5
Moisture (g)	36.3	38.0	37.5	39.7
Carbohydrate, by difference (g)	49.0	48.4	47.5	50.0

**Notes**: USYP, bread containing 80% wheat flour and 20% untreated pea flour (no Revtech process); RT0%, bread containing 80% wheat flour and 20% pea flour Revtech processed at 140 °C with no steam; RT10%, bread containing 80% wheat flour and 20% pea flour Revtech processed at 140 °C with 10% steam; 100%W, bread containing 100% wheat flour (control). * Available carbohydrate = [(total starch × 1.1) − (resistant starch × 1.1) + (total disaccharides × 1.05) + total monosaccharide] [73].

**Table 2:** Demographic characteristics of study subjects.

Baseline Characteristics	Female (n = 12)		Male (n = 12)	
	Mean (SD) ^1^	Range	Mean (SD) ^1^	Range
Age (years)	25 (3.8)	19–31	24 (4.7)	18–34
BMI (kg/m^2^)	22.4 (1.7)	19.6–26.0	22.4 (1.1)	21.0–23.9
Body fat (%)	27.7 (5.6)	18.3–37.1	15.1 (6.1)	3.1–24.1
Waist circumference (cm)	76.0 (5.6)	69.0–89.5	80.1 (4.6)	72.5–86.5
Systolic blood pressure (mm Hg)	102 (5.8)	91–113	108 (8.4)	92–120
Diastolic blood pressure (mm Hg)	70 (5.6)	62–83	70 (8.0)	57–84
**Ethnicity**	**Female (n = 12)**		**Male (n = 12)**	
American/Canadian	8		7	
European	1		2	
Asian	1		2	
Latin	1		0	
Africa	1		0	
Other	0		1	

**Notes:**^1^ SD, standard deviation.

**Table 3:** Postprandial glucose and insulin response (iAUC) in healthy adults, and glycemic index (GI) in different bread treatments.

	Source of Variation (*F*-Value ^3^)						Mean Values ^4^ (Bread Treatments)				Mean Values ^5^ (Sex)	
	Treatment (T)	Sex (S)	Order ^6^ (O)	ID ^7^	T × S	T × O	100%W	USYP	RT0%	RT10%	Male	Female
Glucose iAUC ^1^ (mmol/L × min)	0.64 NS	15.89 ***	11.82 ***	5.65 ***	1.02 NS	0.47 NS	173.04 (15.98)	162.76 (13.60)	160.79 (13.46)	154.70 (16.11)	143.83 ^b^ (11.16)	181.82 ^a^ (8.81)
Insulin iAUC ^1^ (pmol/L × min)	2.01 NS	43.21 ***	28.35 ***	15.04 ***	0.19 NS	0.47 NS	24,295.50 (2784.99)	24,746.08 (2940.76)	26,133.13 (2727.84)	28,507.00 (3604.68)	21,534.58 ^b^ (1972.17)	30,306.27 ^a^ (2102.44)
Glycemic index ^2^	0.39 NS	2.33 NS	2.25 NS	2.97 **	0.40 NS	0.33 NS	-	74.69 (7.78)	69.90 (7.14)	67.78 (6.11)	65.78 (5.27)	75.79 (6.05)

**Notes:** Treatments were USYP, bread containing 80% wheat flour and 20% untreated pea flour (no Revtech process); RT0%, bread containing 80% wheat flour and 20% pea flour Revtech processed at 140 °C with no steam; RT10%, bread containing 80% wheat flour and 20% pea flour Revtech processed at 140 °C with 10% steam; 100%W, bread containing 100% wheat flour (control). ^1^ Incremental areas under the curve (iAUC) for blood glucose and plasma insulin calculated over 120 min time periods. ^2^ Glycemic index = [(glucose response to test food/glucose response to 100%W) × 100 × 0.7]. ^3^ NS, not significant *p* ≥ 0.05; ∗∗ *p* < 0.01; ∗∗∗ *p* < 0.001. ^ab^ Mean values in the same row with different letters are significantly different from each other (*p* < 0.05). ^4^ All values are means (followed in brackets by the standard error), n = 24 participants/treatment. ^5^ All values are means (followed in brackets by the standard error), n = 12 participants/sex. ^6^ Order, order of serving the bread treatments randomly in different visits. ^7^ ID, individual participants.

**Table 4:** Postprandial subjective appetite response (iAUC) in healthy adults following the consumption of bread treatments.

Appetite Sensation Variables ^1^	Source of Variation (*F*-Value ^2^)						Mean Values ^3^ (Bread Treatments)				Mean Values ^4^ (Sex)	
	Treatment (T)	Sex (S)	Order ^5^ (O)	ID ^6^	T × S	T × O	100%W	USYP	RT0%	RT10%	Male	Female
Hunger iAUC (cm × min)	4.02 *	66.88 ***	10.78 ***	16.02 ***	0.78 NS	0.95 NS	609.13 ^a^ (46.27)	509.13 ^b^ (41.34)	547.75 ^ab^ (51.89)	553.19 ^ab^ (45.90)	638.78 ^a^ (29.45)	470.81 ^b^ (31.67)
Fullness iAUC (cm × min)	3.13 *	16.58 ***	12.44 ***	17.09 ***	0.25 NS	1.12 NS	500.13 ^b^ (48.40)	591.13 ^a^ (43.18)	552.19 ^ab^ (47.84)	553.94 ^ab^ (46.76)	506.28 ^b^ (28.92)	592.41 ^a^ (35.41)
Desire to eat iAUC (cm × min)	4.58 *	79.86 ***	9.47 ***	17.10 ***	1.31 NS	2.03 NS	618.88 ^a^ (48.45)	515.06 ^b^ (43.40)	550.06 ^ab^ (51.18)	564.13 ^ab^ (46.95)	652.13 ^a^ (29.10)	471.94 ^b^ (32.90)
Prospective food consumption iAUC (cm × min)	5.93 *	106.71 ***	23.11 ***	17.73 ***	2.26 NS	1.40 NS	603.13 ^a^ (49.37)	494.50 ^b^ (41.50)	528.00 ^b^ (52.53)	531.56 ^b^ (45.91)	636.25 ^a^ (29.67)	442.34 ^b^ (31.54)

**Notes:** Treatments were USYP, bread containing 80% wheat flour and 20% untreated pea flour (no Revtech process); RT 0%, bread containing 80% wheat flour and 20% pea flour Revtech processed at 140 °C with no steam; RT10%, bread containing 80% wheat flour and 20% pea flour Revtech processed at 140 °C with 10% steam; 100%W, bread containing 100% wheat flour (control). ^1^ Total area under the curve (iAUC, cm×min) for appetite sensation variables calculated over 120 min time periods. ^2^ NS, not significant *p* ≥ 0.05; ∗ *p* < 0.05; ∗∗∗ *p* < 0.001. ^ab^ Mean values in the same row with different letters are significantly different from each other (*p* < 0.05). ^3^ All values are means (followed in brackets by the standard error), n = 24 participants/treatment.^4^ All values are means (followed in brackets by the standard error), n = 12 participants/sex. ^5^ Order, order of serving the bread treatments randomly in different visits. ^6^ ID, individual participants.

**Table 5:** Postprandial sensory evaluation and overall acceptability of bread treatments in healthy adults.

Sensory Attributes	Mean Acceptability Values ^3^				*F*-Value ^4^
	100%W	USYP	RT0%	RT10%	
Aroma ^1^	6.6 (0.2)	6.3 (0.2)	6.1 (0.2)	6.5 (0.3)	0.82 NS
Flavor ^1^	7.0 (0.3)	6.6 (0.3)	6.3 (0.3)	6.5 (0.3)	0.78 NS
Color ^1^	6.6 (0.3)	6.3 (0.3)	5.9 (0.3)	6.4 (0.3)	1.22 NS
Texture ^1^	7.3 ^a^ (0.3)	6.8 ^ab^ (0.3)	5.7 ^b^ (0.4)	7.0 ^a^ (0.3)	5.33 **
Overall Acceptability ^1^	6.9 (0.3)	6.7 (0.3)	6.1 (0.3)	6.5 (0.4)	1.17 NS
Frequency of Eating ^2^	6.3 (0.3)	6.0 (0.4)	5.4 (0.4)	5.9 (0.3)	1.09 NS

**Notes:** Treatments were USYP, bread containing 80% wheat flour and 20% untreated pea flour (no Revtech process); RT 0%, bread containing 80% wheat flour and 20% pea flour Revtech processed at 140 °C with no steam; RT10%, bread containing 80% wheat flour and 20% pea flour Revtech processed at 140 °C with 10% steam; 100%W, bread containing 100% wheat flour (control). ^1^ 1 = dislike extremely; 2 = dislike very much; 3 = dislike moderately; 4 = dislike slightly; 5 = neither like nor dislike; 6 = like slightly; 7 = like moderately; 8 = like very much; 9 = like extremely. ^2^ 1 = I would eat this only if forced; 2 = I would eat this if there were no other food choices; 3 = I would hardly ever eat this; 4 = I don’t like this but would eat it on occasion; 5 = I would eat this if available but would not go out of my way; 6 = I like this and would eat it now and then; 7 = I would frequently eat this; 8 = I would eat this very often; 9 = I would eat this every opportunity I had. ^3^ All values are means (followed in brackets by the standard error), n = 24. ^4^ NS, not significant *p* ≥ 0.05; ∗∗ *p* < 0.01. ^ab^ Mean values in the same row with different letters are significantly different from each other (*p* < 0.05).

## Data Availability

Not applicable.

## Supplementary Materials

The following supporting information can be downloaded at: https://www.mdpi.com/article/10.3390/foods11071002/s1, Figure S1: Postprandial glucose response in healthy male (n = 12) and female (n = 12) participants as affected by time (0 to 120 minutes). Figure S2: Postprandial glucose response in healthy male (n = 12) and female (n = 12) participants as affected by bread types. Figure S3: Postprandial insulin response in healthy male (n = 12) and female (n = 12) participants as affected by time (0 to 120 minutes). Figure S4: Postprandial insulin response in healthy male (n = 12) and female (n = 12) participants as affected by bread types. Postprandial glucose and insulin responses in healthy male and female participants as affected by time and bread treatments.
