# Supplementary material for: Acute Effects of Split Pea-Enriched White Pan Bread on Postprandial Glycemic and Satiety Responses in Healthy Volunteers—A Randomized Crossover Trial
Source: Foods. 2022 Mar 29;11(7):1002. doi: 10.3390/foods11071002 (PMC8997531; doi:10.3390/foods11071002)
Supplement: Supplementary file 1 [file foods-11-01002-s001.zip › foods-1582489-supplementary.pdf]

Supplementary Materials

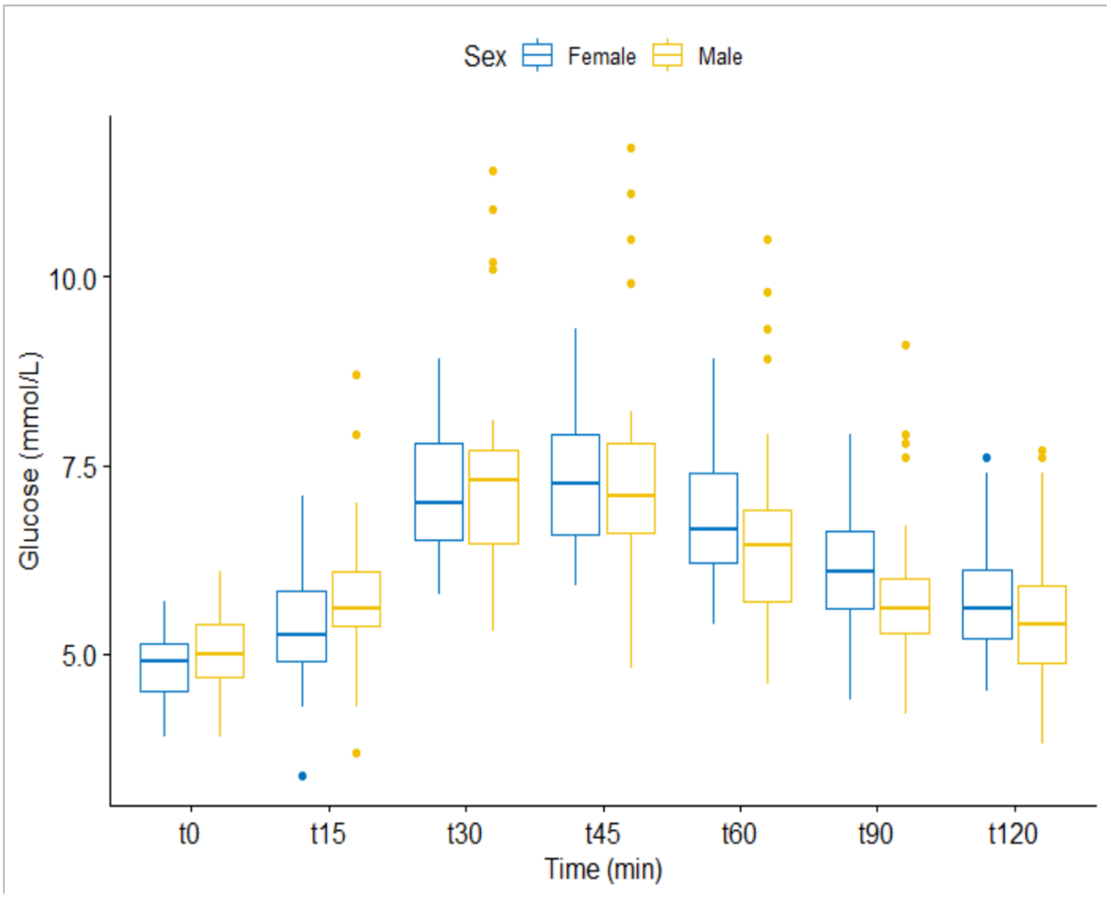

Figure S1: Postprandial glucose response in healthy male (n = 12) and female (n = 12) participants as affected by time (0 to 120 minutes).

Values are means (n=12 participants/ sex), with their standard errors represented by vertical bars.

Notes: Treatments were **100%W**, bread containing 100% wheat flour (control); **USYP**, bread containing 80% wheat flour and 20% untreated pea flour (no Revtech Process); **RT0%**, bread containing 80% wheat flour and 20% pea flour Revtech Process 140°C with no steam; **RT10%**, bread containing 80% wheat flour and 20% pea flour Revtech Process 140°C with 10% steam.

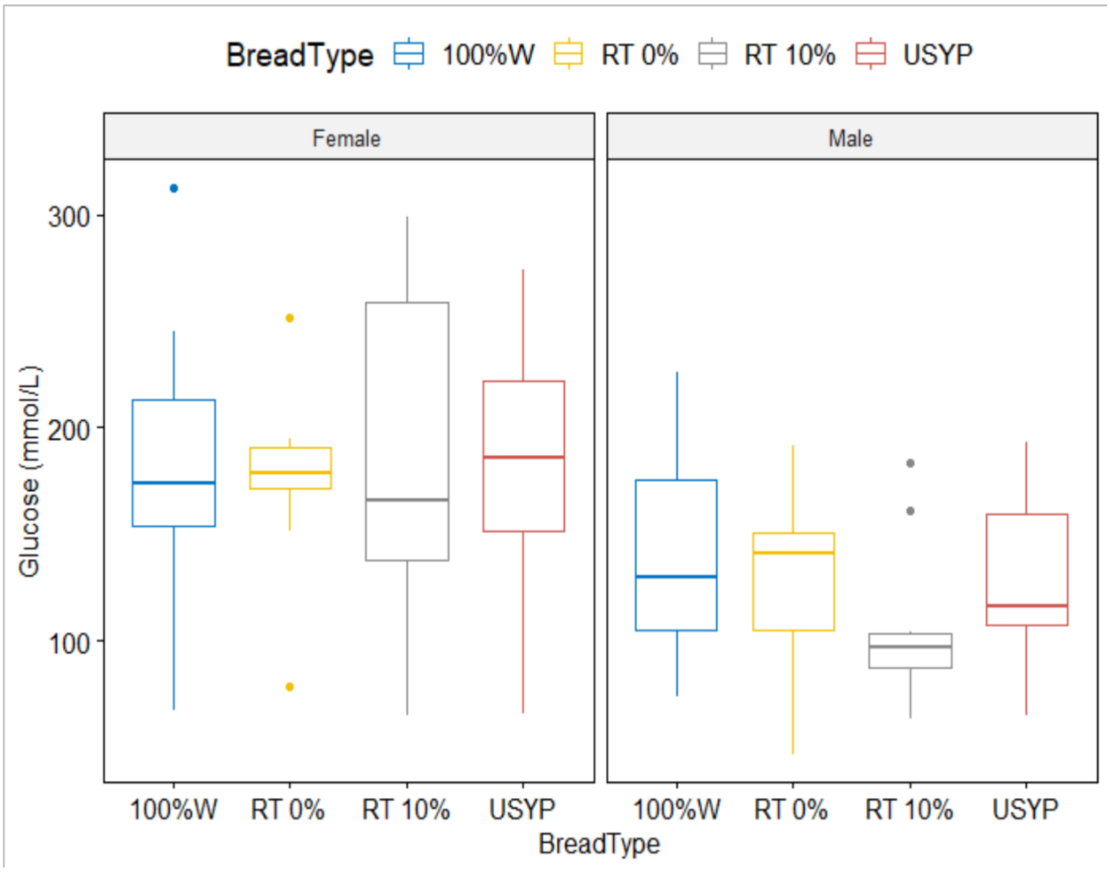

Figure S2: Postprandial glucose response in healthy male (n = 12) and female (n = 12) participants as affected by bread types.

Values are means (n=24 participants/treatment), with their standard errors represented by vertical bars.

Notes: Treatments were **100%W**, bread containing 100% wheat flour (control); **USYP**, bread containing 80% wheat flour and 20% untreated pea flour (no Revtech Process); **RT0%**, bread containing 80% wheat flour and 20% pea flour Revtech Process 140°C with no steam; **RT10%**, bread containing 80% wheat flour and 20% pea flour Revtech Process 140°C with 10% steam.

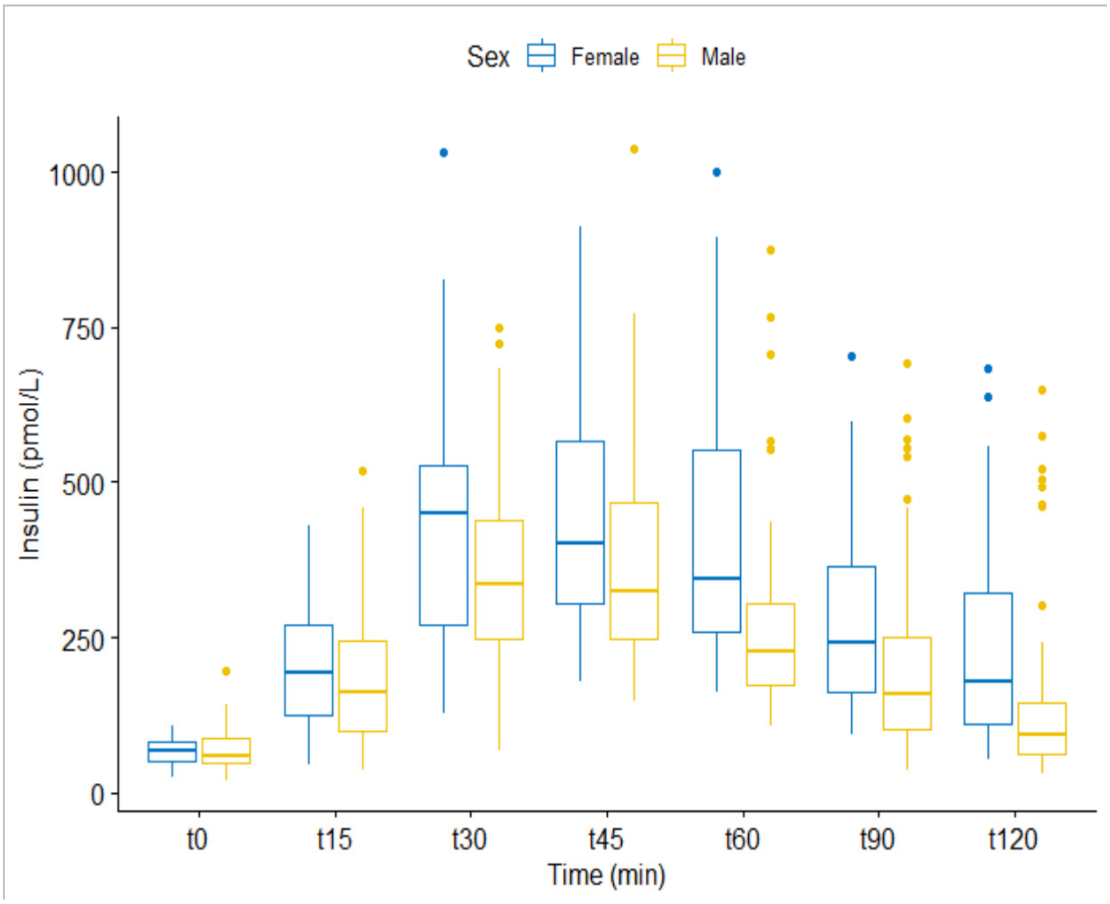

Figure S3: Postprandial insulin response in healthy male (n = 12) and female (n = 12) participants as affected by time (0 to 120 minutes).

Values are means (n=12 participants/ sex), with their standard errors represented by vertical bars.

Notes: Treatments were **100%W**, bread containing 100% wheat flour (control); **USYP**, bread containing 80% wheat flour and 20% untreated pea flour (no Revtech Process); **RT0%**, bread containing 80% wheat flour and 20% pea flour Revtech Process 140°C with no steam; **RT10%**, bread containing 80% wheat flour and 20% pea flour Revtech Process 140°C with 10% steam.

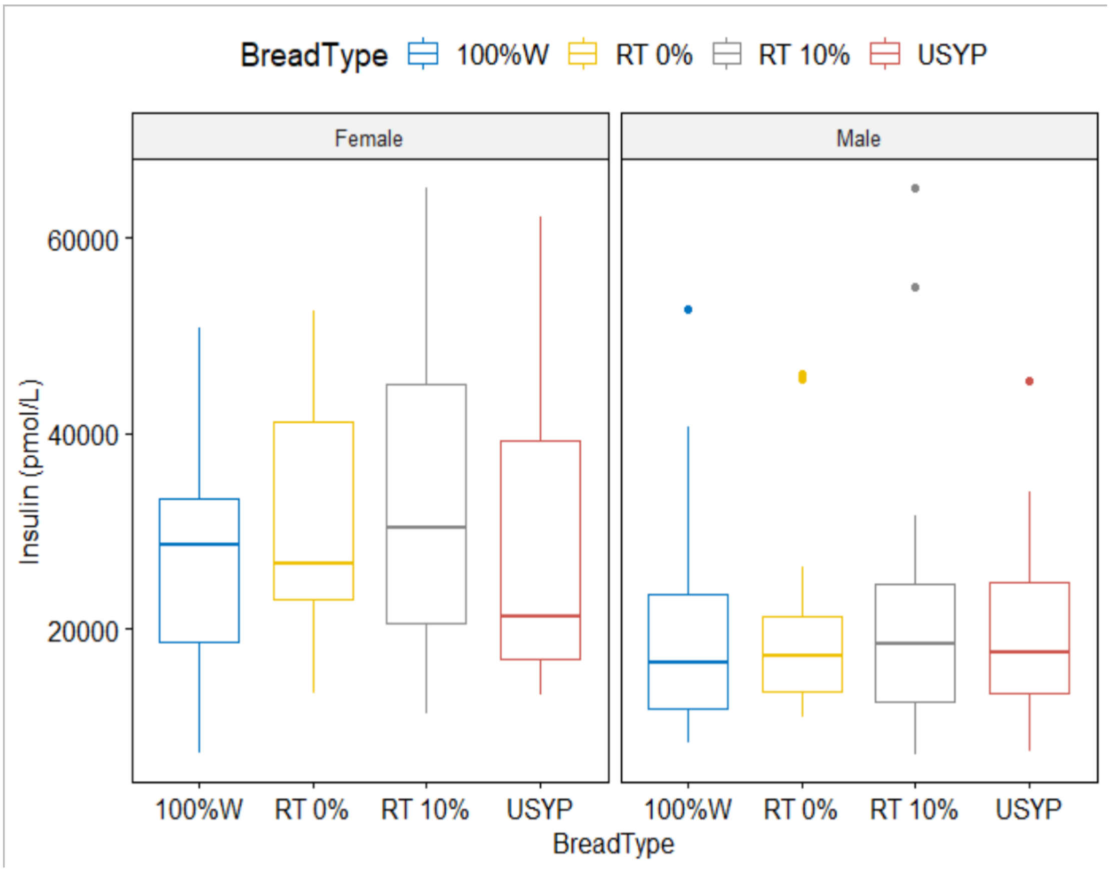

Figure S4: Postprandial insulin response in healthy male (n = 12) and female (n = 12) participants as affected by bread types.

Values are means (n=24 participants/treatment), with their standard errors represented by vertical bars.

Notes: Treatments were **100%W**, bread containing 100% wheat flour (control); **USYP**, bread containing 80% wheat flour and 20% untreated pea flour (no Revtech Process); **RT0%**, bread containing 80% wheat flour and 20% pea flour Revtech Process 140°C with no steam; **RT10%**, bread containing 80% wheat flour and 20% pea flour Revtech Process 140°C with 10% steam.
